# Suprascapular glomus tumor: an unusual cause of chronic shoulder pain

**DOI:** 10.1080/23320885.2021.2014335

**Published:** 2021-12-10

**Authors:** Theodoros Floros, Konstantinos Perivoliotis, Athina Samara, Aikaterini Tsionga, Maria Ioannou, Ioannis Baloyiannis, Konstantinos Tepetes

**Affiliations:** a Department of Surgery, University Hospital of Larissa, Larissa, Greece; b Panepistemio Thessalias Tmema Iatrikes, University of Thessaly, Larissa, Greece; c Department of Urology, General Hospital of Larissa, Larissa, Greece; d Department of Pathology, University Hospital of Larissa, Larissa, Greece

**Keywords:** Suprascapular, extradigital, glomus, tumor, shoulder

## Abstract

Glomus tumors (GT) are rare mesenchymal tumors that develop in the subungual digital region. Extradigital GTs are very rare, with atypical clinical features. We report the case of a 63-year-old male with a five-year history of intermittent shoulder pain, where an excisional biopsy confirmed the diagnosis of a glomus tumor.

## Introduction

Glomus tumors (GT) are rare neoplasms, representing less than 2% of all soft tissue tumors [1]. GTs originate from the glomus apparatus, a component of the reticular dermis [1]. It consists of a specialized arteriovenous shunt, surrounded by connective tissue and glomus cells, and has a pivotal role in thermoregulation [2,3]. Although cases of malignant transformation have been reported, GTs, primarily, display a benign behavior [4].

GTs are typically located in the upper extremities, with almost 75% of them developing in the subungual region of the digits [4,5]. Typical presentation includes a solitary nodule associated with intermittent debilitating pain crises, localized tenderness and temperature sensitivity [4].

Although extradigital GTs have been reported in the literature, they are considered quite infrequent, with atypical clinical features, resulting to markedly therapeutic delays [6]. More specifically, the suprascapular anatomical area is among the least common extradigital site of GTs [7]. The rarity of these tumors, alongside the heterogeneity in the presenting symptoms, render suprascapular GTs a diagnostic challenge [7]. Therefore, the prolonged time until final diagnosis, allows GTs to be masked under the clinical manifestation of chronic shoulder pain [7].

Herein, we report a rare case of an extradigital GT, located in the left suprascapular area, and manifesting as chronic shoulder pain.

## Case presentation

A 63-year-old male presented to the outpatient department of our institution with a five-year history of pain in the left supra-scapular area. The symptoms were paroxysmal and became incapacitating during the last year. The patient noted that even the application of cold water and minimal direct stimulation triggered the onset of pain. He previously sought treatment with local steroidal ointments, oral non-steroidal anti-inflammatory drugs (NSAIDs) and physiotherapy with little to no success. His BMI was 38 kg/m^2^, while his past medical history included hypertension, coronary heart disease, diabetes, COPD, and hyperuricemia.

Physical examination revealed a small reddish nodule in the left supra-scapular area. The lesion was immobile, well-defined and with no associated inflammatory signs. Upon palpation, pin-point tenderness was recorded. There were no pathological findings from both neurological and vascular clinical examination. Similarly, no palpable axillary and cervical lymph nodes were noted.

**Figure 1.**
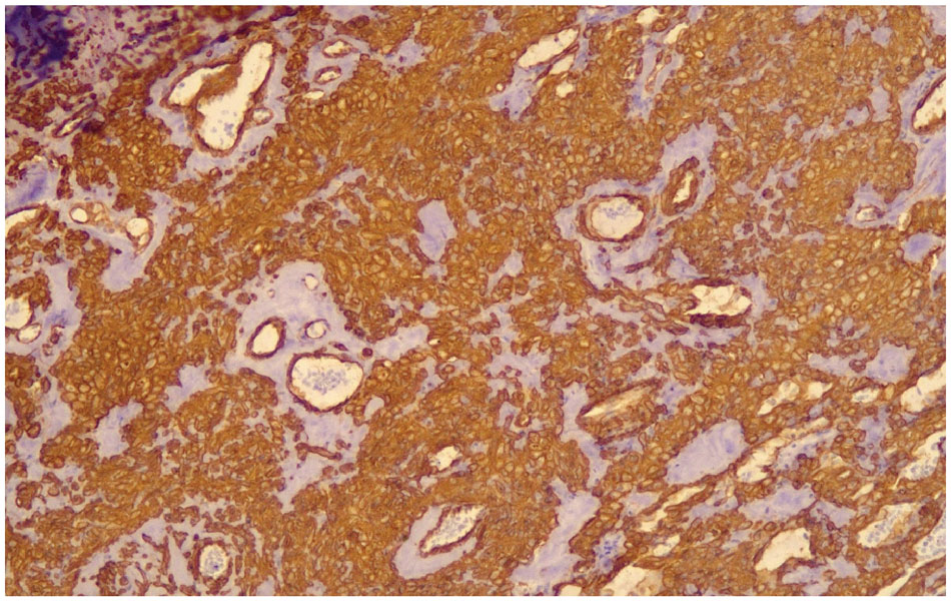
Glomus tumor forming a circumscribed mass, consisting of uniform cells (haematoxylin and eosin stain, original magnification ×10).

**Figure 2.**
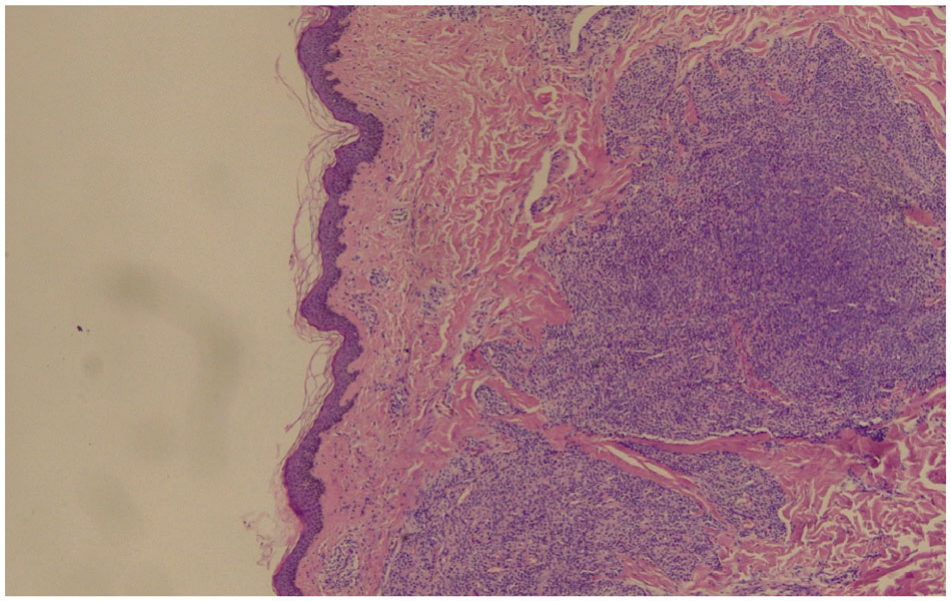
Monomorphic tumor cells with centrally placed, round nuclei, are arranged in perivascular nests. The vessel wall shows marked hyalinization. (haematoxylin and eosin stain, original magnification ×20).

**Figure 3.**
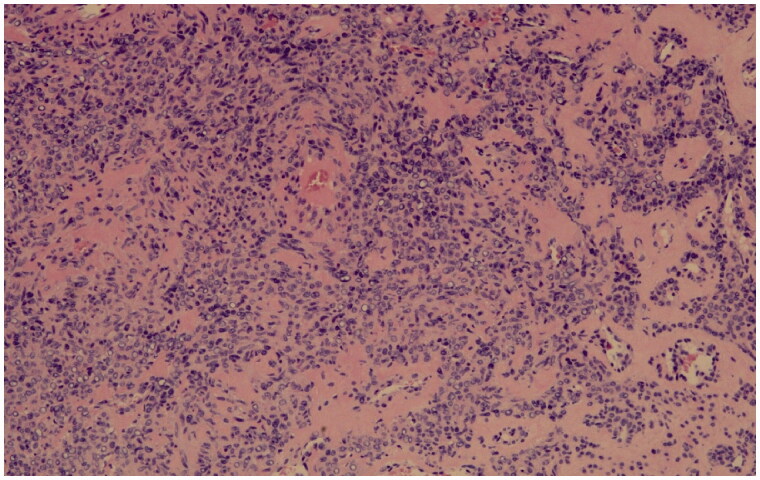
The tumor cells are strongly positive for smooth muscle actin (SMA immunostain, original magnification ×10).

An excisional biopsy, with wide resection margins was performed, revealing a tumor of a size about 1.6 × 1 × 0.6 cm. The histopathologic examination a revealed a circumscribed mass consisting of uniform cells in haematoxylin eosin stain (Figure 1). A negative resection margin was confirmed. The tumor cells had centrally placed nuclei and were arranged in perivascular nests. A marked hyalinization was identified in the tumoral vessel walls (Figure 2). Immunostaining for smooth muscle actin (SMA), vimentin and caldesmon was strongly positive, whereas a negative result for CKAE1/AE3, S100 and MELANA was noted. Positive CD34 loci were also identified. Based on these, the diagnosis of a glomus tumor was confirmed (Figure 3).

On the seventh post-operative day, wound dehiscence was noted, treated with regular wound dressings care. A complete secondary intention wound healing was achieved twenty days after excision. At six months follow-up, a total remission of symptoms was confirmed, with no signs of local or distant recurrence.

## Discussion

Glomus tumors are rare mesenchymal neoplasms arising from the glomus body [8]. GTs typically develop between the 3rd and the 5th decade of life [9]. Due to the higher density in glomus bodies, the subungual area of the digits, is the most common anatomical area of GT development [10]. Extradigital GTs, though, are quite uncommon, consisting only a 26.7% rate of all new diagnoses [10]. Regarding gender allocation, subungual tumors are associated with a 2:1 female predominance, while extradigital GTs are more frequently diagnosed in male patients [10].

Glomus apparatus is a specialized neuro-myo-arterial unit located in the dermo-hypodermic junction [4]. It consists of multiple arterio-venous shunts that form a complex vascular network, also known as Sucquet-Hoyer canals [4]. Temperature changes, as sensed by nerve fibers within the glomus body, result to the contraction of the covering globus cells, thus providing thermoregulatory functional capabilities to this structure [4].

The exact pathogenetic origin of extradigital GTs though, is, still, unclear [11]. Several theories regarding the oncogenesis of GTs have been suggested [11]. Initial reports proposed that GTs were either hyperplasias, or heterotopic proliferations of glomus cells deriving from the glomus body [11]. However, glomocytic differentiation of perivascular cells is, currently, considered as the most accepted explanatory theory of GTs pathogenesis [11]. The latter, also, explains the morphological similarities between GTs and other perivascular mesenchymal tumors [11].

Although GT is considered as a benign neoplasia, cases of malignant transformation and distal metastases have been reported [12]. Malignancy should be considered in deep tumors with a diameter larger than 2 cm, combined with atypical mitoses, and moderate to high nuclear grade and mitotic activity (5 MFs/50 HPFs) [12].

Typical manifestation of GTs is a solitary tumor associated with intermittent pain crises, triggered by alterations of temperature and pressure gradient [11]. The pain intensity is remarkably variable and often irradiates to the limbs and the trunk [11]. Thus, the classic diagnostic triad of GTs includes paroxysmal pain, localized tenderness, and cold hypersensitivity [13]. Moreover, regional hyperesthesia, muscle atrophy and osteopenia can be, also, noted in some cases [13]. Several clinical examinations have been introduced as a means to facilitate diagnosis, including the Love test (point tenderness), the Posner test (cold induced pain) and the Hildreth test (ischemia test) [13].

However, only a small percentage of extradigital GTs present with the typical clinical triad [5]; in fact, most of them display atypical clinical features [5]. Lee et al. [14], compared digital and extradigital GTs and concluded that pain and cold sensitivity rates were significantly lower in the latter. Furthermore, in the cohort by Schiefer et al. [15], only 2% of extradigital tumors cold precipitated pain crises. These were also confirmed in a recent review by Vieira et al. [7], regarding GTs in the wider anatomical area of the shoulder. Interestingly, our patient displayed the full clinical symptomatology, a rare phenomenon for suprascapular GTs. Despite these, due to the atypical lesion location, the initial differential diagnosis extended on multiple painful skin tumors (e.g. dermatofibroma, schwannoma, and leiomyoma) [16].

The atypical presenting symptoms and the low diagnostic accuracy of the clinical tests, often leads to misdiagnoses and delays in the delivery of the optimal treatment [4]. Literature reports confirm that the average duration of symptoms is seven years and in some cases can extend up to 30 years [17]. This symptom chronicity poses a considerable health and socioeconomic issue since it is related with ineffective treatments and a direct impact on patients’ physical and psychological functionality [7]. In our case, the patient was symptomatic for 5 years, during which he received several analgesic schemes, with no apparent effect on his quality of life.

Imaging has an eminent role as a diagnostic adjunct. Magnetic resonance imaging (MRI) is the most sensitive imaging study for GT with a sensitivity and specificity of 90%, and 50%, respectively [18]. However, MRI, in almost one third of cases is non-diagnostic; especially in pathologically and anatomically atypical tumors [8]. Additionally, given its availability and low cost, ultrasonography can be used for the diagnosis and characterization of extradigital GTs [19].

Surgical excision remains the standard of care resulting to the immediate resolution of the symptoms and the minimization of recurrence risk [15]. Previous series reported that after GT resection a 10–30% recurrence rate should be expected [9]. However, in a review by Geramizadeh et al. [20], none of the GTs in the shoulder area had a recurrence during the postoperative follow up. Although the follow up period of our patient was minimal when compared to the respective literature, no recurrence signs were found.

The present study reported the rare case of a suprascapular GT presenting with chronic shoulder pain symptoms. Increased clinical suspicion regarding extradigital GTs is required to reduce misdiagnosis rates, and the physical and mental consequences of delayed diagnoses.

## Data Availability

Data available on request from the authors
